# Spatiotemporal Change and the Natural–Human Driving Processes of a Megacity’s Coastal Blue Carbon Storage

**DOI:** 10.3390/ijerph18168879

**Published:** 2021-08-23

**Authors:** Wenbo Cai, Qing Zhu, Meitian Chen, Yongli Cai

**Affiliations:** 1 School of Design & China Institute for Urban Governance, Shanghai Jiao Tong University, Shanghai 200240, China; wbcai@rcees.ac.cn (W.C.); jzzc0504@163.com (Q.Z.); 2 School of Ecological and Environmental Sciences, East China Normal University, Shanghai 200241, China; meitianchen@126.com; 3 State Key Laboratory of Urban and Regional Ecology, Research Center for Eco-Environment Sciences, Chinese Academy of Sciences, Beijing 100085, China

**Keywords:** land use/land cover, spatiotemporal dynamics, driving forces, blue carbon, coastal zone, megacity, urban governance

## Abstract

Coastal blue carbon storage (CBCS) plays a key role in addressing global climate change and realizing regional carbon neutrality. Although blue carbon has been studied for some years, there is little understanding of the influence of a megacity’s complex natural and human-driven processes on CBCS. Taking the Shanghai coastal area as an example, this study investigated the spatiotemporal change in CBCS using the InVEST (Integrated Valuation of Ecosystem Services and Tradeoffs) model during 1990–2015, and analyzed the response of the CBCS to a megacity’s complex natural- and human-driven processes through a land use/land cover transition matrix and hierarchical clustering. The results were as follows: (1) Thirty-three driving processes were identified in the study area, including four natural processes (e.g., accretion, succession, erosion, etc.), two human processes (reclamation and restoration) and twenty-seven natural–human coupled processes; they were further combined into single and multiple processes with positive and negative influences on the CBCS into four types (Mono+, Mono−, Multiple+ and Multiple− driving processes). (2) Shanghai’s CBCS increased from 1659.44 × 10^4^ Mg to 1789.78 ×10^4^ Mg, though the amount of Shanghai’s coastal carbon sequestration showed a decreasing trend in three periods: 51.28 × 10^4^ Mg in 1990–2000, 42.90 × 10^4^ Mg in 2000–2009 and 36.15 × 10^4^ Mg in 2009–2015, respectively. (3) There were three kinds of spatiotemporal patterns in the CBCS of this study area: high adjacent to the territorial land, low adjacent to the offshore waters in 1990; high in the central part, low in the peripheral areas in 2009 and 2015; and a mixed pattern in 2000. These patterns resulted from the different driving processes present in the different years. This study could serve as a blueprint for restoring and maintaining the CBCS of a megacity, to help mitigate the conflicts between socioeconomic development and the conservation of the CBCS, especially in the Shanghai coastal area.

## 1. Introduction

With the growth of the global population and urban expansion, social demand for economic growth and degradation of ecosystems are on the rise [1,2]. Since human activities and climate change are putting greater pressure on the marine ecosystem, there has been an increasing awareness of the value and potential of marine carbon storage in tackling global climate change [3,4,5]. The concept of ‘blue carbon’ has been widely accepted. ‘Blue carbon’ refers to the process, activities and mechanisms of using marine or coastal ecosystems to absorb and sequestrate CO_2_ from the atmosphere [6]. Compared with a forestry carbon sink, a blue carbon sink has a higher carbon sequestration rate and higher carbon density [6,7,8]. To cope with global climate change and realize regional carbon neutrality, it is necessary to explore blue carbon storage and sequestration.

Most of the previous research on carbon storage focused on terrestrial ecosystems (especially forest ecosystems) [9,10,11]. However, recent studies revealed that coastal ecosystems, as an ecological buffer zone connecting land and sea, has huge carbon storage potential and its carbon sequestration capacity per unit area was far greater than that of terrestrial carbon sinks [6,7,8]. For example, coastal wetlands, as an important type of coastal ecosystem, had a carbon sequestration rate 15 times that of the terrestrial ecosystem, thus playing an irreplaceable role in mitigating global warming and bringing social and economic benefits [7]. Therefore, the exploration, conservation and promotion of carbon storage and sequestration of coastal ecosystems, one of the ‘nature-based solutions’, will be helpful to achieve the goal of regional carbon neutrality [12,13,14].

The rapid growth of the population and the demand for fast economic growth have led to dramatic changes in land use/land cover (LULC) in coastal areas globally, which will definitely affect the distribution and change of coastal blue carbon storage (CBCS). Specifically, coastal erosion, beach reclamation, seawall construction and other land use-driven processes have released huge amounts of carbon dioxide in coastal areas, making the coastal ecosystem gradually converted into a huge carbon source [7,15]. On the other hand, land use-driven processes, such as beach succession and swamp restoration, also helped carbon dioxide storage in soil and vegetation, and continuously enhanced the CBCS capacity of coastal ecosystems [16,17,18]. Previous studies showed that the LULC change caused by natural or human-driven processes had both positive and negative effects on coastal blue carbon [13,15,19]. However, few studies had been conducted on the interrelations between CBCS and the natural and human-driven processes that generated the LULC changes [8]. Tang et al. (2018) had pointed out that clarifying the response and adaptation mechanism of CBCS to natural changes and human activities helped enhance people’s scientific comprehension of the mechanism of increasing CBCS [7]. A systematic understanding of the key driving processes of LULC change that affect the CBCS can provide more reasonable land use strategies for governments to effectively reduce and mitigate the negative impact on blue carbon sequestration.

In a coastal megacity, CBCS has been significantly influenced by the great changes in LULC driven by complex natural and human-driven processes, e.g., accretion and reclamation [13,20]. Although CBCS has been studied for some years, there is little understanding of the influence of a megacity’s complex natural and human-driven processes on CBCS [8]. Shanghai, located in the east of China, is a coastal megacity. The coastal area of Shanghai was not only an important carbon sink, but also played an important role in maintaining biodiversity, improving the ecological environment and ensuring the sustainable development of the local society and economy [21]. However, its coastal ecosystems have been influenced by natural and human-driven processes, e.g., accretion and reclamation [21]. This study takes the Shanghai coastal area as an example to investigate the influence of a megacity’s natural and human-driven processes on CBCS. The specific objectives of this study are to (1) identify the spatiotemporal pattern and analyze the conversion of LULC in Shanghai coastal areas in 1990, 2000, 2009 and 2015; (2) analyze the dynamics and identify the spatiotemporal pattern of CBCS in Shanghai coastal areas in 1990, 2000, 2009 and 2015; and (3) analyze the natural and human-driven processes and put forward coastal land management strategies for Shanghai’s coastal areas. The purpose of this study is to deepen people’s understanding of the relationship between LULC change, the natural–human driving processes and CBCS, thus providing a reference for coastal land management strategies.

## 2. Materials and Methods

### 2.1. Study Area

**Figure 1:**
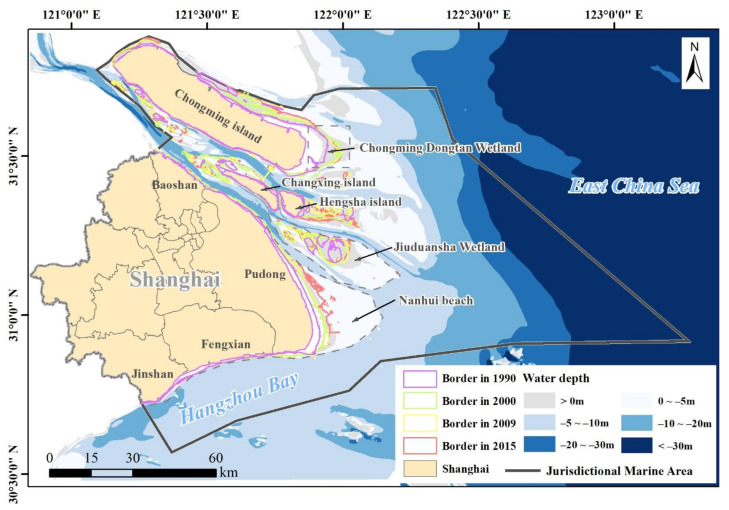
The location and scope of the study area.

The study area (30°37′ N to 30°54′ N, 121°16′ E to 123°00′ E) is located in the north of the East China Sea, adjacent to the mainland in the west, and next to the waters of the Zhejiang and Jiangsu provinces in the south and north (Figure 1). It includes the coastal zone and the offshore waters and has a total area of 11,231.32 km^2^ and continental coastline of about 211 km^2^, as described in the Marine Functional Zoning of Shanghai (2011–2020) (Figure 1). The coastal zone is located at the Yangtze River Estuary, including the coastal area of Chongming Island, the tidal flats of Changxing Island and Hengsha Island, Jiuduansha Wetland, the beaches of Nanhui District, Fenxian District and Jinshan District. The internal border of the coastal zone is the coastal line of 1990 (Figure 1). The external border of the coastal zone changed, being pink in 1990, green in 2000, yellow in 2009 and red in 2015, corresponding to the expansion of the coastal area (Figure 1). The offshore waters include most of the Yangtze River Estuary, a small part of Hangzhou Bay and part of the East China Sea.

The study area is characterized by a subtropical monsoon climate with abundant rainfall. The annual average temperature is between 15 °C and 17 °C, and the annual precipitation is between 1000 mm and 1800 mm [12]. The dominant species include two native species (*Phragmites australis* and *Scripus mariqueter*) and one invasive species (*Spartina alterniflora*) [22]. The study area has natural coastal wetlands with fertile soil (formed by the huge amount of sediment carried by the runoff of the Yangtze River and deposited at the interface of the river and the sea), which is a potentially an important land resource. Chongming Dongtan Wetland is a national nature reserve for birds and an internationally important Ramsar wetland [18]. Jiuduansha Wetland is also a national nature reserve [18]. As a megacity in China, Shanghai has high carbon dioxide emissions from human activities. In addition, urban expansion reduces the coastal wetland area [23], leading to a decline in the CBCS as a result.

### 2.2. Database and LULC Classification

We used Landsat TM images data collected from the U.S. Geological Survey (USGS) (http://www.usgs.gov/ (accessed on 1 February 2019)) to generate the land use and land cover (LULC) classification maps of 1990, 2000, 2009 and 2015. The resolution of the Landsat TM imagery was 30 m and these data were primary processed through Level 1 Product Generation System (LPGS), which included systematic radiometric and geometric corrections. In order to improve the accuracy, we did an extensive manual editing after auto classification by referencing the very-high-resolution imagery and the historical materials. The overall accuracy of the thematic maps in 1990, 2000, 2009 and 2015 were over 85%. The LULC type is shown in Table 1.

### 2.3. LULC Transition Matrix and Classifiction of the Land Use/Land Cover Driving Processes

The LULC transition matrix tables were set up as 1990–2015, 1990–2000, 2000–2009 and 2009–2015 in ArcGIS v10.5. Based on the LULC transition matrix and previous studies [8,19], the driving processes were classified into six types (Table 2), which were accretion (A), succession (S), regressive succession (Rs), erosion (E), reclamation (R) and restoration (Re). Accretion, succession and restoration are positive processes, while erosion, regressive succession and reclamation are negative processes.

### 2.4. Calculation of Carbon Storage and Sequestration

The carbon storage module of the InVEST (Integrated Valuation of Ecosystem Services and Tradeoffs) model, version 3.3.1 (https://naturalcapitalproject.stanford.edu/software/invest (accessed on 1 February 2019)), combine the LULC change with the dynamic transformation of carbon storage in space to assess the ecosystem carbon storage [13,15,24]. The calculation formula is as follows:

(1) $$C_{i}=C_{i\_\mathrm{above}}+C_{i\_\mathrm{below}}+C_{i\_\mathrm{dead}}+C_{i\_\mathrm{soil}}$$

(2) $$C_{tot}=\sum_{i=1}^{n}C_{i}\times S_{i}$$

where *i* is a type of LULC; *C_i_* is the carbon storage of LULC type *i* (Mg/ha); *C_i_*__above_ is the aboveground carbon storage of LULC type *i* (Mg/hm^2^); *C_i_*__ below_ is the belowground carbon storage of LULC type *i* (Mg/hm^2^); *C_i_*__ dead_ is the dead organic carbon storage (Mg/ha) of LULC type I; *C_i_*__ soil_ is the carbon storage (Mg/ha) of soil with soil use type I; *C_tot_* is the total carbon storage (Mg) of the ecosystem; *S_i_* is the area of LULC type *i* (ha); and *n* is the number of LULC types, with the *n* in this paper being 10.

The InVEST model’s required inputs for the module calculation are simple, universal and stable [13,15,24]. The inputs included the current and future LULC files, and an editable carbon pools table of carbon density. The outputs include a total current carbon storage map and future carbon storage map, and a carbon sequestration map: the difference between the carbon stored currently and in the future (https://storage.googleapis.com/releases.naturalcapitalproject.org/invest-userguide/latest/carbonstorage.html (accessed on 1 February 2019)).

The carbon density of each LULC types was defined as per previous studies [13,16,17,20] (Table 3), based on the principles of regional similarity and data availability. This study defined that the CBCS was composed of the CBCS of the coastal zone and the CBCS of the offshore waters. The carbon density of each LULC type in the study area was divided into four levels (Highest, Higher, Lower, Lowest) by a natural break in ArcGIS.

### 2.5. Hierarchical Clustering

In order to analyze the influence of the different driving processes on the change in CBCS, we used R v4.0 to call the ‘pheatmap’ package library (R package Version 1.0.12) [25] to show the distribution of the CBCS values. In this case study, the CBCS in relation to the driving processes in 1990, 2000, 2009 and 2015 were displayed using a Clustered Heat Map (CHM). The CHM of the CBCS dynamic was generated by using the ‘pheatmap’ package in R Version 3.1.3 (www.r-project.org/ (accessed on 1 February 2019)).

(1) We calculated the accumulated amount of coastal blue carbon storage (CSCS) of each driving process in each year (1990, 2000, 2009 and 2015), and then all the values of the CBCS were transformed into a 33 × 4 matrix (the amount of CBCS of the 33 driving processes in 1990, 2000, 2009 and 2015 was calculated in the matrix);
(2) We normalized the values of the 33 CBCS samples under specific driving processes in each period by Z score transformation, in which the Z score was calculated as (X − μ)/σ (X is the input value of CBCS, μ is the average value of 33 CBCS samples and σ is the standard deviation of the CBCS values);
(3) All the normalized values of CBCS were used to generate the CHM of the CBCS dynamics in the heatmap function in the R ‘pheatmap’ package. The hierarchical clustering algorithm was used in the R ‘pheatmap’ package to measure the similarities of CBCS within the heat map by calculating the Euclidean distance [8].

## 3. Results

### 3.1. LULC Changes in Shanghai Coastal Area

**Figure 2:**
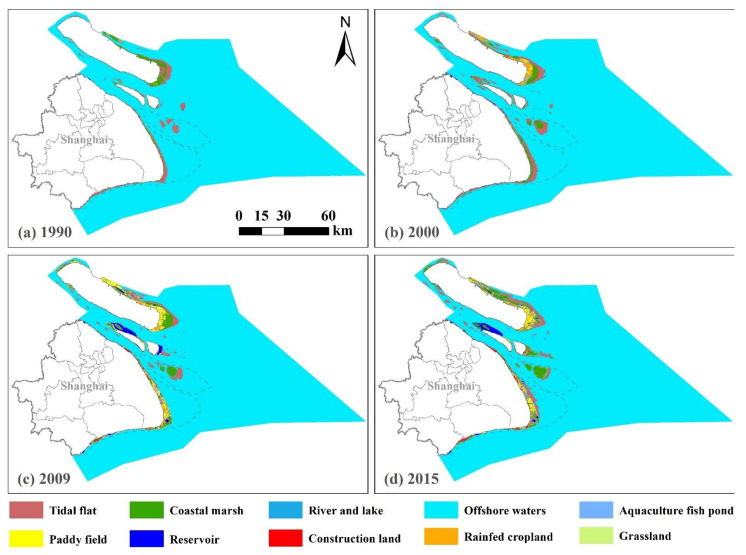
The distribution of the land use/land cover types along the Shanghai coastal area from 1990 to 2015. (**a**) The spatial distribution of land use/land cover types in the study area in 1990. (**b**) The spatial distribution of land use/land covers in the study area in 2000. (**c**) The spatial distribution of land use/land cover types in the study area in 2009. (**d**) The spatial distribution of land use/land cover types in the study area in 2015.

There were significant spatial differences in LULC changes in the coastal zone during 1990–2015. The LULC types of the coastal zone adjacent to the territorial land changed greatly over time, while there was little change in the LULC types of the coastal zone adjacent to the offshore waters. Figure 2 illustrated that the tidal flats and coastal marshes showed a concentrated and contiguous distribution in the east coast of Shanghai, especially in Chongming Island and Jiuduansha Wetlands in 1990. Then, a fragmentated pattern appeared in these types in Chongming Island, while they expanded in the east coast of Pudong District, Changing Islands and Jiuduansha Wetlands in 2000. Finally, a fragmented pattern appeared in the east coast of Pudong District, while they continuously expanded in the east coast of Pudong District and Jiuduansha Wetlands in 2009 and 2015. The aquaculture fish ponds and construction lands were scattered sparsely in the coastal zone adjacent to the territorial land in 1990 and 2000, and gradually dispersed in patches on the coastline of Jinshan Beach and Fengxian Beach, the west of Changxing Island and the east of Hengsha Island in 2009 and 2015. The paddy fields and rainfed cropland slowly changed from having a sporadic distribution in 1990 to a banded distribution in 2000. Then, these areas further expanded and showed a concentrated and contiguous distribution in 2009 and 2015. The reservoirs mainly appeared in the west of Changxing Island and east of Hengsha Island in 2009 and disappeared in the east Hengsah Island in 2015.

The conversion between the offshore waters and LULC types in the coastal zone occurred with the conversion of natural wetlands and artificial LULC types simultaneously during 1990–2015 (Table 4). The offshore waters had the largest transfer-out areas. Table 4 indicate that 469 km^2^ of the offshore waters were converted into other LULC types, in particular 159 km^2^ into tidal flats. Among the three natural wetland types, the tidal flats had the largest transfer area of 249.50 km^2^, which were mainly converted into coastal marshes (transfer area is 76.17 km^2^). Furthermore, 112.11 km^2^ of the coastal marshes were mainly converted into paddy fields, rainfed cropland and aquaculture fish ponds; the transfer-out area of rivers and lakes was the least, only 0.13 km^2^. Regarding the artificial wetland type, there was a large transfer-out of aquaculture fish ponds, with an area of 24.02 km^2^, which was mainly restored to coastal marsh, with an area of 8.39 km^2^. Among the other land types, rainfed cropland had the largest transfer-out area (12.46 km^2^). 

Table 4 indicates that the transfer-in/out types and amount of LULC types varied across the three periods (1990–2000, 2000–2009 and 2009–2015), with co-occurrence of the two main LULC conversion process during 1990–2015. From 1990–2000, offshore waters had the largest transfer-out area (321.05 km^2^), which was mainly converted into tidal flats (248.49 km^2^), coastal marshes (36.31 km^2^) and aquaculture fish ponds (22.39 km^2^). In the coastal zone, the tidal flats had the largest transfer-out area (180.67 km^2^), which was mainly converted into coastal marshes (88.42 km^2^), aquaculture fish ponds (19.12 km^2^), rainfed cropland (18.67 km^2^) and paddy fields (15.77 km^2^), besides conversion into offshore waters (33.45 km^2^). From 2000 to 2009, the tidal flats in the coastal zone had the largest transfer-out area (253.12 km^2^), which was mainly converted into coastal marshes (81.66 km^2^) and aquaculture fish ponds (81.66 km^2^), besides conversion into offshore water (66.72 km^2^). The offshore waters had the second largest transfer-out area (218.94 km^2^), which was converted into tidal flats (128.47 km^2^), coastal marshes (14.58 km^2^), and reservoirs (49.82 km^2^). The transfer-in area of the reservoir increased dramatically in this period. From 2009 to 2015, the offshore waters had the largest transfer-out area (189.61 km^2^), which was mainly converted into tidal flats (148.24 km^2^) and coastal marshes (20.22 km^2^). In the coastal zone, the tide flats had the second largest transfer-out area (150.68 km^2^), which was mainly converted into coastal marshes (53.78 km^2^) and aquaculture fish ponds (21.78 km^2^), besides conversion into offshore waters (54.25 km^2^). 

### 3.2. Spatiotemporal Change in CBCS in the Shanghai Coastal Area

**Figure 3:**
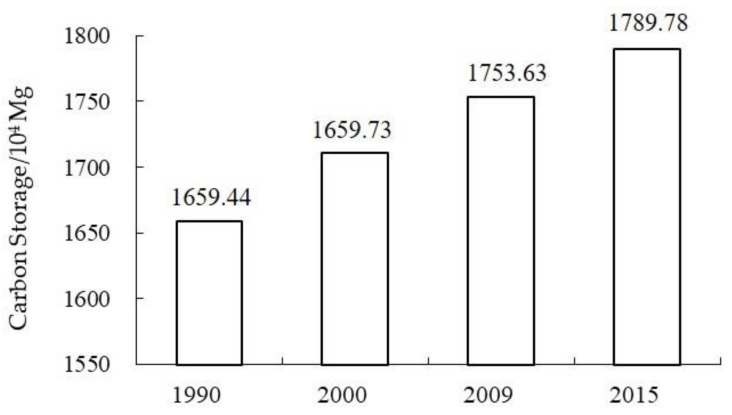
The dynamics of blue carbon storage in the Shanghai coastal area during 1990–2015.

The total amount of CBCS increased from 1659.44 × 10^4^ Mg to 1789.78 ×10^4^ Mg during 1990–2015 (Figure 3), though the amount of Shanghai’s coastal carbon sequestration showed a decreasing trend in the three periods: 51.28 × 10^4^ Mg in 1990–2000, 42.90 × 10^4^ Mg in 2000–2009 and 36.15 × 10^4^ Mg in 2009–2015, respectively. The total amount of carbon sequestration was 130.48 × 10^4^ Mg from 1990 to 2015.

**Figure 4:**
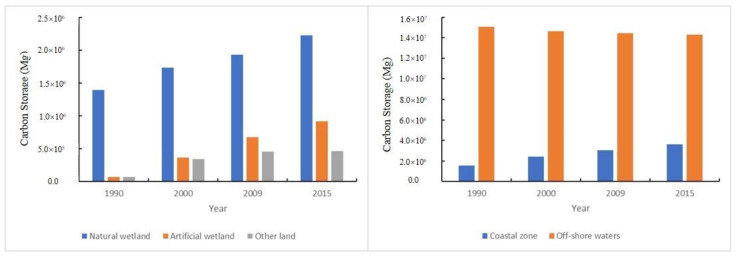
Comparison of the blue carbon storage of natual wetlands, artificial wetlands and other land types in the Shanghai coastal area during 1990–2015.

The CBCS in natural wetlands, artificial wetlands and other lands showed an upward trend during 1990–2015, with a different growth rate in the three periods (1990–2000, 2000–2009 and 2009–2015) (Figure 4). The CBCS of the artificial wetlands and the other lands increased dramatically, with a growth rate of 422.45% and 37.3.82%, respectively, during 1990–2000, after which their growth rates decreased to 85.35% and 35.92%, respectively, during 2000–2009, and finally their growth rates decreased to 34.71% and 1.56%, respectively. The natural wetlands had a relatively constant growth rate (average 17.01%) during 1990–2015. The CBCS of the coastal zone showed an upward trend with an average growth rate (average 25.12%) during 1990–2015, while the CBCS of the offshore waters remained relatively stable, with only a slightly negative growth rate (average −1.28%).

**Figure 5:**
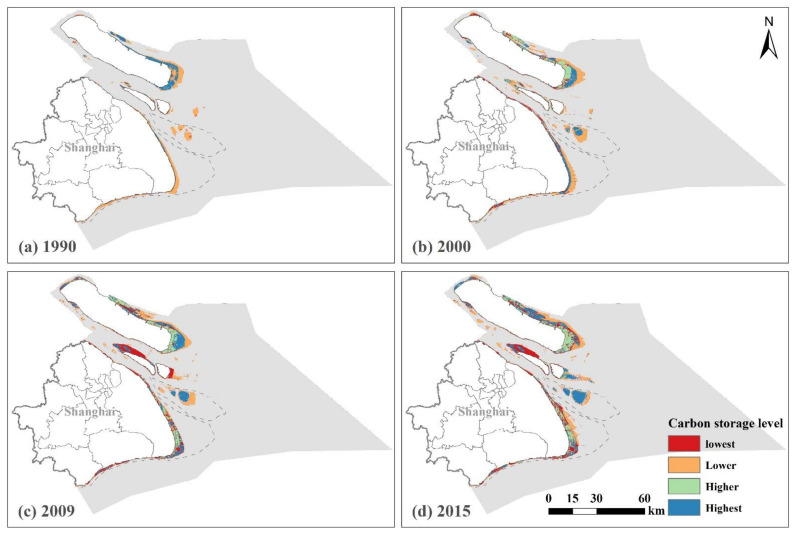
The spatiotemporal distribution of the four levels (Highest, Higher, Lower and Lowest) of blue carbon storage in the Shanghai coastal area during 1990–2015. (**a**) The spatial distribution of the four levels of coastal blue carbon storage in the study area in 1990. (**b**) The spatial distribution of the four levels of coastal blue carbon storage in the study area in 2000. (**c**) The spatial distribution of the four levels of coastal blue carbon storage in the study area in 2009. (**d**) The spatial distribution of the four levels of coastal blue carbon storage in the study area in 2015.

There were different spatial patterns in the four carbon storage levels in the different years. The Highest areas were distributed in the coastal zone adjacent to the territorial land, while the Lower areas were distributed in the coastal zone adjacent to the offshore areas in 1990 (Figure 5). The Lower areas showed a concentrated and contiguous distribution in the peripheral areas of the coastal zone, while the Highest areas were mainly concentrated in the interior of Chongming Island in 1990.

In 2000, the Lower areas were distributed in the peripheral areas of the coastal zone (east coast of Pudong Districit, Changing Islands, Hengsha Island and Jiuduansha Wetlands), while the interior areas of Chongmin Island were occupied by the Higher areas. The Highest areas showed a line pattern in the interior of the east coast of Shanghai, which were mainly located between the High areas and Lower areas in Chongming Island, the central area of the Jiuduansha Wetlands and east coast of Changxing Island during this period.

In 2009, the Lowest areas appeared and were concentrated in the west coast of Changxing Island and east coast of Hengsha Island. The Lower areas were continuously distributed in the peripheral areas of the coastal zone, while the Higher areas largely expaned into the east coast of Shanghai (e.g., east of Pudong District). In 2015, the Highest areas were distributed in the central part of the coastal zone, while the Lower areas were distributed in the peripheral part of the coastal zone, which was similar to the previous period, except that the Highest areas expanded into the Jiuduansha Wetlands and east coast of Changxing Island, but declined in the east coast of Chongmin Island although expanding into its the north coast.

### 3.3. Driving Processes of CBCS in the Shanghai Coastal Area

#### 3.3.1. Influence of Different Driving Processes on the Change of Blue Carbon Storage

This study identified thirty-three driving processes (Table 5). From the perspective of a positive change in CBCS, the driving process of A-S and A accounted for the largest proportion of the total blue carbon increase, 25.28% and 20.87%, respectively. However, the change range of CBCS per unit area was quite different. A-S and A were 49.67 Mg/ha and 13.17 Mg/ha for 25 years, respectively. This indicated that the positive driving process after natural accretion (e.g., A-S) was more conducive to an increase in CBCS per unit area. 

From the perspective of a negative change in CBCS, the driving process of R (65.78%), E (14.90%) and Rs-R (8.32%) had a great negative impact on CBCS in the study area (Table 5), among which Rs-R had a greater impact on the CBCS per unit area. The other six driving processes (R-Re-R, Rs, Rs-E, E-R, Rs-S-R and E-A-E) had a relatively small impact on the reduction in CBCS, but Rs-E and Rs had a large change impact on CBCS per unit area, which were −50.01 Mg/ha and −43.74 Mg/ha, respectively. It can be seen that Rs, Rs-R or Rs-E played a key role in the change in CBCS per unit area. 

**Figure 6:**
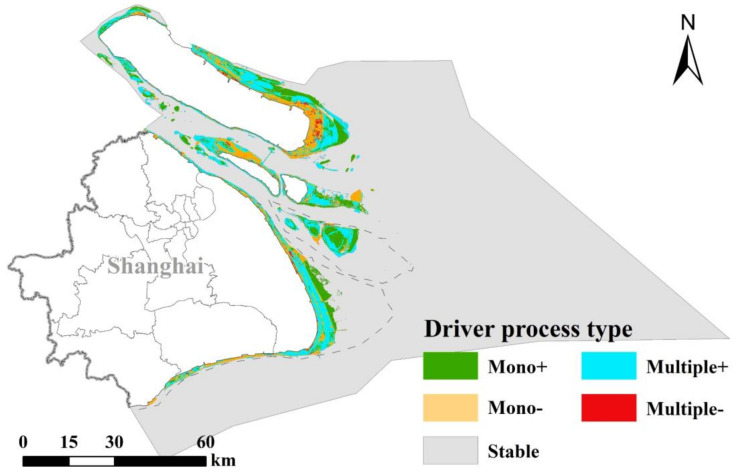
The spatial distribution of the four driving processes during 1990–2015. Note: Mono+: increase in carbon storage by a single driving process; Mono−: decrease in carbon storage by a single driving process; Multiple+: increase in carbon storage by multiple driving process: Multiple−: decrease in carbon storage by multiple driving process; Stable: no change in carbon storage and land cover. For example, A-E-A indicates accumulation of multiple influences (‘Accretion–Erosion–Accretion’) on carbon storage across the 25 years.

The thirty driving processes were classified into four types based on the direction of the driving process on carbon storage: single driving process of carbon storage increase (Mono+), single driving process of carbon storage decrease (Mono−), multiple driving process of carbon storage increase (Multiple+) and multiple driver carbon stock reduction process (Multiple−). Specifically, the ‘Mono+’ or ‘Mono−’ type indicated that the change in carbon storage was only affected by a single driving process, including natural or human factors. The ‘Multiple+’ or ‘Multi–’ types indicated that the change of carbon storage was accumulated by multiple driving processes (Table 5). The ‘Mono+’ type areas and ‘Multiple+’ type areas showed a concentrated and contiguous distribution in the peripheral areas of the coastal zone, while the ‘Mono+’ and ‘Multiple−’ were mainly concentrated in the interior of Chongming Island and west coast of Changxing Island (Figure 6).

#### 3.3.2. The Clustering of Driving Processes of Blue Carbon Storage

**Figure 7:**
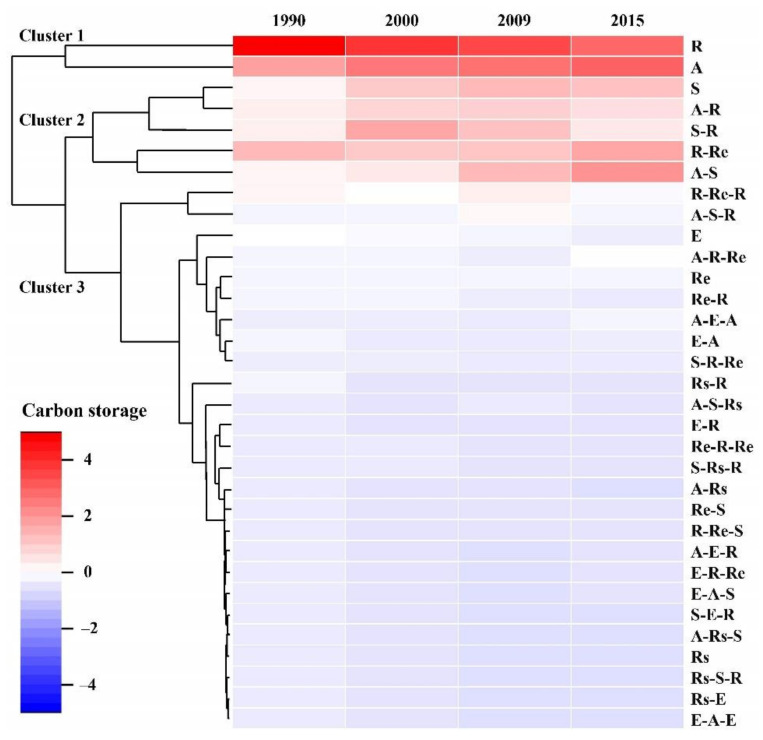
The clustering analysis of blue carbon storage driven by the major processes in the Shanghai coastal area. Note: Driving Process—A: Accretion; S: Succession; Rs: Regressive succession; E: Erosion; R: Reclamation; Re: Restoration. For example, R-Re-R means that the area was under reclamation between 1990 and 2000, restored between 2000 and 2009 and then again under reclamation between 2009 and 2015.

Figure 7 indicates the changes in blue carbon storage as influenced by the thirty-three types of driving processes. The clustering results show the amount of the carbon storage related to the driving processes in the different years. The first clustering group indicates that R and A drove the changes in LULC types, with high CBCS (>2) from 1990 to 2015. R reduced the CBCS, while A increased the CBCS. The second clustering group indicates that S, A-R, S-R, R-Re and A-S drove the change in LULC types, with a higher CBCS (0~2) from 1990 to 2015. These driving processes increased the CBCS. The second clustering group indicated that twenty-six driving processes drove the change in LULC types, with a low (<−2) and lower CBCS (−2~0) from 1990 to 2015. These had little influence on the CBCS.

## 4. Discussion

### 4.1. Spatiotemporal Change and Driving Processes of CBCS

Previous studies often focused on the influence of one kind of driving process in the study area, such as reclamation [13,22,26], and often ignored the influence of multiple coupled natural and human driving processes. In this study, we found that CBCS increased from 1990 to 2015, though the coastal carbon sequestration continuously decreased, due to the interactions between the natural driving processes (e.g., accretion) and human-driven processes (e.g., reclamation). The accumulated positive influence of the natural processes (e.g., accretion) exceeded the accumulated negative influence of the human-induced processes, yet the negative influence of the human-induced processes (e.g., disturbing the reclamation of coastal marshes) was strengthened during the study period. 

The location where natural and human-driven processes took place may be different from each other in the same period. We found three different patterns in CBCS across the coastal zone: high adjacent to the territorial land, low adjacent to the offshore waters in 1990; high in the central part, low in the peripheral areas in 2009 and 2015; and a mixed pattern in 2000. These spatial patterns were formed by natural and human-driven processes at different locations in each period. For example, accretion and other natural processes were mainly on the coastal zone adjacent to the territorial land, while reclamation and other human-driven processes were mainly on the coastal zone adjacent to the offshore waters.

The natural and human-driven processes that took a dominant role in forming the spatial pattern of the CBCS varied across the different periods. The natrual driving processes drove the spatial pattern of the CBCS in 1990. The spatial pattern of the CBCS was mainly influenced by the distribution of the coastal mash and tidal flats in 1990. The spatial pattern of certain areas was influenced by human activities in 2000, including agriculture and aquaculture activities. The spatial pattern of certain areas was influenced by negative human activities in 2009 and 2015, e.g., urbanization in Nanhui beach, and positive human activities, e.g., restoration in Chongming Island and Jiuduansha Wetlands.

A combination of natural and human-driven processes may be different from the combination of a single driving process, leading to more types of complex influences. Thirty-three driving processes were identified in Shanghai’s coastal area, including four natural processes (e.g., accretion, succession, erosion, etc.), two human processes (reclamation and restoration) and twenty-seven human–natural coupled processes; they were further classified into four types: Mono+, Mono−, Multiple+ and Multiple− driving processes. 

A combination of positive natural driving processes and negative human-driven processes may lead to an increase in CBCS. For example, a combination of reclamation and restoration may reduce the negative impact of reclamation. In the negative human-driven processes of CBCS, reclamation had the most negative impact on the CBCS, which indicated that the negative impact of reclamation on CBCS cannot be ignored [13,27]. Previous studies indicated that reclamation had become one of the most widespread threats to coastal wetlands, which seriously affected the structure, function and service of coastal ecosystems [28]. In positive human-driven processes, restoration had an impact on the change in blue carbon storage, which also reflected that the ecological restoration project under human intervention could effectively weaken the negative impact of reclamation on CBCS [7,29].

Based on the results of this study, we put forward coastal LULC management strategies for CBCS in the Shanghai coastal area as follows: first, the driving processes that have a large negative influence on CBCS in the study area, whether they be single or multiple driving processes, should be restricted by land-controlling polices. For example, some types of LULC conversion induced by reclamation (R) or its combination process should be controlled or forbidden, as they have huge negative impacts on CBCS, e.g., coastal marshes into construction land. Second, some positive single or combination processes should be encouraged in the coastal area, e.g., restoration (Re) and combination processes with positive influences. Restoration policies should be strengthened for wetland restoration, such as coastal marshes, especially for wetland ecosystems that has regressive succession or that were influenced by reclamation. Moreover, a combination of negative driving processes with positive processes is suggested to be a useful way for CBCS conservation, e.g., a combination of reclamation and restoration, and combination of reclamation and succession.

### 4.2. Contributions and Limitations

In this study, we identified the spatiotemporal pattern of CBCS in the Shanghai coastal area, which was influenced by natural–human driving processes. We systematically classified the types of driving processes (human, natural and natural–human coupled driving processes; single and multiple driving processes), and analyzed the influence of these driving process models on the CBCS. An immediate change in carbon storage after land use change is assumed in this study. However, the change in storage to a new steady state can take many decades. Carbon accumulation rates in different biomes can be measured directly (e.g., by eddy covariance, EC) or indirectly (by repeated carbon inventories). In particular, there are eddy covariance sites in wetlands (including coastal), paddy fields and other ecosystems mentioned in the study, in particular in China. In addition, the assessment of the CBCS was mainly based on the coastal ecosystem types, and not the quality of the coastal ecosystems, which we will further study in the future.

## 5. Conclusions

This study explored spatiotemporal change in CBCS, using the InVEST model, along the Shanghai coast during 1990–2015, and analyzed the effect this megacity’s complex natural and human-driven processes have on CBCS. The major findings are summarized as follows. First, Shanghai’s CBCS increased, though the amount of Shanghai’s coastal carbon sequestration showed a decreasing trend in 1990–2000, 2000–2009 and 2009–2015. Second, we found three kinds of spatiotemporal patterns in the CBCS of this study area: high adjacent to the territorial land, low adjacent to the offshore waters in 1990; high in the central part, low in the peripheral areas in 2009 and 2015; and a mixed pattern in 2000. Third, we identified thirty-three driving processes in the study area, including four natural processes, two human-induced processes and twenty-seven natural–human coupled processes; they were further classified into four types (Mono+, Mono−, Multiple+ and Multiple− driving processes). Based on the results of this study, the combination of negative driving processes with positive processes is suggested to be a useful way for CBCS conservation, e.g., reclamation–restoration. This study could serve as a blueprint for restoring and maintaining CBCS in a megacity, to mitigate the conflicts between socioeconomic development and the conservation of CBCS, especially for the Shanghai coastal area.

## Figures and Tables

**Table 1:** Land use/land cover classification.

First Class	Second Class	Description
Natural wetland	Tidal flat	A silty or sandy shoal with no vegetation cover located in the intertidal zone
	Coastal marsh	Any of various bogs and mire areas located on the coast and in the intertidal zone
	River and lake	Inland permanent waters
Artificial wetland	Aquaculture fish pond	Coastal zone of farming area
	Paddy field	Arable land where rice is grown
	Reservoir	A man-made water storage area
Other land	Construction land	Impervious water surface or man-made structure
	Rainfed cropland	Seasonally unplanted arable land
	Grassland	Low vegetation or grassland
Waters	Offshore waters	Permanent waters within the jurisdictional area

**Table 2:** Driving processes and the initial–final land use/land cover types in the Shanghai coastal area.

Class	Driving Process	Ecological Consequence	Initial–Final Land Use/Land Cover (LULC) Types
Natural Driving Process	Accretion (A)	Growing tidal flats by sand deposition around estuarine area	1-2, 1-3, 5-2, 5-3
	Succession (S)	Natural development of estuarine ecosystem from pioneer (e.g., tidal wetlands) to the local climax ecosystem (brackish & freshwater marsh)	2-3, 2-4, 3-4
	Regressive succession (Rs)	Ecosystem degradation from climax to pioneer ecosystem in estuarine area	3-2
	Erosion (E)	Loss in tidal wetlands by seawater washing or seal level rise	2-1, 3-1, 4-1
Human-Driven Processes	Reclamation (R)	Loss in coastal wetlands by encroachment of land reclamation	(1, 2, 3, 4, 5)-6, (1, 2, 3, 4, 5)-7, (1, 2, 3, 4, 5)-8, (1, 2, 3, 4, 5)-9, (1, 2, 3, 4, 5)-10
	Restoration (Re)	Increased area or enhanced ecological functioning of coastal wetland by human intervention	(6, 7, 8, 9, 10)-1, (6, 7, 8, 9, 10)-2, (6, 7, 8, 9, 10)-3, (6, 7, 8, 9, 10)-4, (6, 7, 8, 9, 10)-5

Note: 1: Offshore waters; 2: Tidal flat; 3: Coastal marsh; 4: Grassland; 5: River and lake; 6: Aquaculture fish pond; 7: Paddy field; 8: Reservoir; 9: Construction land; 10: Rainfed cropland. For example, 1-2 means transition from land use/land cover (LULC) type 1 to type 2.

**Table 3:** Carbon density of the land use/land cover types in the Shanghai coastal area (unit: Mg/ha).

Land Use/Land Cover	*C_i__*_above_	*C_i__*_below_	*C_i__*_soil_	*C_i__*_dead_	*C_tot_*
Tidal flat	2.8	1.8	13.8	0.0	18.4
Offshore water	2.0	1.0	11.0	0.0	14.0
Coastal marsh	26.5	10.5	26.6	0.4	32.4
River and lake	1.5	0.5	11.0	0.0	13.0
Aquaculture fish pond	0.5	0.0	12.0	0.0	12.5
Paddy field	9.0	4.0	38.6	0.3	25.5
Reservoir	1.0	0.5	11.0	0.0	12.5
Construction land	0.0	0.0	8.0	0.0	8.0
Rainfed cropland	5.0	4.0	31.7	0.3	20.5
Grassland	2.5	11.1	26.5	0.2	40.3

Note: Carbon density was mainly referenced from [13,16,17,20].

**Table 4:** The land use/land cover transition matrix of the Shanghai coastal area (unit: km^2^).

2015											
1990	Grassland	Offshore Waters	Rivers and Lakes	Construction Land	Paddy Fields	Reservoir	Tidal Flat	Rainfed Cropland	Coastal Marsh	Aquaculture Fish Ponds	Transfer-Out Area
Grassland	0.00	0.08	0.00	0.22	0.28	0.06	0.02	0.36	0.51	0.35	1.88
Offshore waters	1.68	0.00	1.87	14.57	31.21	50.92	158.94	24.56	124.3	61.17	469.22
Rivers and lakes	0.00	0.01	0.00	0.03	0.00	0.00	0.05	0.01	0.01	0.02	0.13
Construction land	0.03	0.02	0.00	0.00	0.01	0.12	0.15	0.37	0.14	0.26	1.10
Paddy fields	0.21	0.12	0.00	0.33	0.00	0.06	0.07	1.33	0.96	1.54	4.62
Reservoir	0.01	0.19	0.02	0.13	0.04	0.00	0.02	0.36	0.10	0.04	0.91
Tidal flat	2.71	33.88	0.29	15.70	45.73	10.41	0.00	27.59	76.17	37.02	249.50
Rainfed cropland	0.78	0.10	0.01	1.23	1.94	0.54	0.72	0.00	4.26	2.88	12.46
Aquaculture fish pond	0.71	0.80	0.02	4.06	4.10	0.53	1.11	4.30	8.39	0.00	24.02
Coastal marsh	5.65	2.73	0.31	6.32	41.63	1.87	2.55	26.65	0.00	24.40	112.11
Transfer-in area	11.78	37.93	2.52	42.59	124.94	64.51	163.63	85.53	214.84	127.68	—
2000											
1990	Grassland	Offshore waters	River and Lakes	Construction land	Paddy Fields	Reservoir	Tide flat	Rainfed Cropland	Aquaculture fish pond	Coastal marshes	Transfer-Out area
Grassland	0.00	0.07	0.01	0.13	0.49	0.02	0.12	0.27	0.54	0.33	1.98
Offshore waters	0.12	0.00	0.01	5.45	7.69	4.61	238.49	5.98	22.39	36.31	321.05
River and lakes	0.00	0.02	0.00	0.01	0.00	0.00	0.04	0.01	0.04	0.01	0.13
Construction land	0.02	0.04	0.00	0.00	0.15	0.07	0.16	0.33	0.27	0.03	1.07
Paddy fields	0.07	0.12	0.02	0.40	0.00	0.04	0.33	1.57	1.37	0.91	4.83
Reservoir	0.00	0.48	0.00	0.18	0.01	0.00	0.22	0.08	0.09	0.01	1.07
Tide flat	0.50	33.45	0.45	3.15	15.77	1.14	0.00	18.67	19.12	88.42	180.67
Rainfed cropland	0.17	0.25	0.02	0.72	2.49	0.17	1.50	0.00	3.55	3.81	12.68
Aquaculture fish pond	0.10	1.12	0.18	1.11	3.70	0.03	4.60	3.55	0.00	6.38	20.77
Coastal marsh	0.82	3.11	0.91	3.27	17.95	0.12	6.82	45.79	24.97	0.00	103.76
Transfer-in area	1.80	38.66	1.60	14.42	48.25	6.20	252.28	76.25	72.34	136.21	—
2009											
2000	Grassland	Offshore waters	River and Lakes	Construction land	Paddy Fields	Reservoir	Tide flat	Rainfed Cropland	Aquaculture fish pond	Coastal marshes	Transfer-Out area
Grassland	0.00	0.00	0.00	0.08	0.85	0.00	0.00	0.37	0.13	0.29	1.72
Offshore waters	0.03	0.00	0.76	5.54	0.94	49.82	128.47	5.47	13.33	14.58	218.94
River and lakes	0.02	0.00	0.00	0.31	0.11	0.00	0.18	0.29	0.29	0.40	1.60
Construction land	0.06	0.14	0.00	0.00	0.32	0.17	1.11	5.3	2.86	0.92	10.88
Paddy fields	0.79	0.78	0.08	2.45	0.00	1.19	1.16	8.56	7.52	16.06	38.59
Reservoir	0.00	1.13	0.00	1.02	0.01	0.00	0.26	0.18	0.42	0.23	3.25
Tide flat	0.50	66.72	1.24	8.56	12.72	22.83	0.00	23.39	35.50	81.66	253.12
Rainfed cropland	1.19	0.35	0.07	2.36	35.86	0.19	2.62	0.00	6.12	9.08	57.84
Aquaculture fish pond	1.29	8.57	0.18	6.22	9.53	2.00	6.23	12.01	0.00	13.48	59.51
Coastal marsh	2.01	4.41	0.12	6.72	14.52	1.01	6.75	21.35	18.96	0.00	75.85
Transfer-in area	5.97	82.10	2.45	33.26	74.86	77.21	146.78	76.92	85.13	136.70	—
2015											
2009	Grassland	Offshore waters	River and Lakes	Construction land	Paddy Fields	Reservoir	Tide flat	Rainfed Cropland	Aquaculture fish pond	Coastal marshes	Transfer-Out area
Grassland	0.00	0.00	0.00	0.21	2.07	0.01	0.02	1.42	0.83	1.06	5.62
Offshore waters	0.07	0.00	1.34	3.44	1.20	2.24	148.24	2.91	9.95	20.22	189.61
River and lakes	0.01	0.05	0.00	0.09	0.50	0.21	0.00	0.19	0.58	0.81	2.44
Construction land	0.14	0.53	0.02	0.00	1.00	0.39	1.98	4.61	5.09	1.90	15.66
Paddy fields	4.95	0.01	0.18	0.81	0.00	0.17	0.24	16.71	9.92	12.26	45.25
Reservoir	0.11	6.28	0.83	0.38	4.53	0.00	2.83	1.38	6.37	7.43	30.14
Tide flat	0.34	54.25	0.59	3.85	6.18	2.45	0.00	7.46	21.78	53.78	150.68
Rainfed cropland	2.72	0.03	0.01	5.46	28.19	0.40	0.80	0.00	15.14	19.36	72.11
Aquaculture fish pond	0.67	1.66	0.31	5.49	12.05	6.92	4.29	9.94	0.00	28.36	69.69
Coastal marsh	3.07	0.61	0.08	4.60	30.63	5.44	8.41	21.16	29.84	0.00	103.84
Transfer-in area	12.08	63.42	3.36	24.33	86.35	18.23	166.81	65.78	99.50	145.18	—

**Table 5:** The variation in blue carbon storage driven by the main processes.

Driving Process	Total (Mg)	Magnitude (Mg/ha)	Categories	Attribute
R	−141,945.05	−6.24	Mono−	Human
E	−32,146.32	−8.82	Mono−	Natural
Rs-R	−17,943.62	−38.66	Multiple−	Natural–Human
R-Re-R	−12,722.48	−4.72	Multiple−	Human
Rs	−2937.84	−43.40	Mono−	Natural
Rs-E	−2749.99	−50.01	Multiple−	Natural
E-R	−2599.15	−6.70	Multiple−	Natural–Human
Rs-S-R	−1519.68	−28.13	Multiple−	Natural–Human
E-A-E	−1211.05	−13.06	Multiple−	Natural
S-E-R	1016.51	24.55	Multiple+	Natural–Human
A-Rs	1104.87	6.60	Multiple+	Natural
A-Rs-S	1287.70	49.51	Multiple+	Natural
A-S-Rs	2252.43	6.60	Multiple+	Natural
A-E-R	2741.35	9.85	Multiple+	Natural–Human
R-Re-S	3009.98	18.04	Multiple+	Natural–Human
E-R-Re	3153.11	21.71	Multiple+	Natural–Human
Re-R	3285.47	2.57	Multiple+	Human
E-A-S	4847.99	41.31	Multiple+	Natural
S-Rs-R	4882.34	18.48	Multiple+	Natural–Human
Re-S	6396.07	35.88	Multiple+	Natural–Human
Re-R-Re	9568.51	33.32	Multiple+	Human
E-A	12,143.60	9.82	Multiple+	Natural
S-R-Re	22,811.57	37.67	Multiple+	Natural–Human
A-S-R	32,424.10	16.66	Multiple+	Natural–Human
Re	38,868.58	29.97	Mono+	Human
A-E-A	46,761.63	26.11	Multiple+	Natural
S-R	58,399.40	10.21	Multiple+	Natural–Human
A-R-Re	82,106.25	41.73	Multiple+	Natural–Human
A-R	102,806.38	11.72	Multiple+	Natural–Human
R-Re	158,546.30	18.12	Multiple+	Human
S	214,231.34	42.52	Mono+	Natural
A	315,009.94	13.17	Mono+	Natural
A-S	381,527.51	49.67	Multiple+	Natural

Note: A: Accretion; S: Succession; Rs: Regressive succession; E: Erosion; R: Reclamation; Re: Restoration.

## Data Availability

The data are contained within the article.
